# Carbon stock of Moso bamboo (*Phyllostachys pubescens*) forests along a latitude gradient in the subtropical region of China

**DOI:** 10.1371/journal.pone.0193024

**Published:** 2018-02-16

**Authors:** Mengjie Xu, Haibao Ji, Shunyao Zhuang

**Affiliations:** 1 College of Public Administration, Nanjing Agricultural University, Nanjing, Jiangsu Province, China; 2 Institute of Subtropical Crops, Zhejiang Academy of Agricultural Sciences, Wenzhou, Zhejiang Province, China; 3 State Key Lab of Soil and Sustainable Agriculture, Institute of Soil Science, Chinese Academy of Sciences, Nanjing, Jiangsu Province, China; Assam University, INDIA

## Abstract

Latitude is an important factor that influences the carbon stock of Moso bamboo (*Phyllostachys pubescens*) forests. Accurate estimation of the carbon stock of Moso bamboo forest can contribute to sufficient evaluation of forests in carbon sequestration worldwide. Nevertheless, the effect of latitude on the carbon stock of Moso bamboo remains unclear. In this study, a field survey with 36 plots of Moso bamboo forests along a latitude gradient was conducted to investigate carbon stock. Results showed that the diameter at breast height (DBH) of Moso bamboo culms increased from 8.37 cm to 10.12 cm that well fitted by Weibull model, whereas the bamboo culm density decreased from 4722 culm ha^−1^ to 3400 culm ha^−1^ with increasing latitude. The bamboo biomass carbon decreased from 60.58 Mg C ha^−1^ to 48.31 Mg C ha^−1^ from north to south. The total carbon stock of Moso bamboo forests, which comprises soil and biomass carbon, ranged from 87.83 Mg C ha^−1^ to 119.5 Mg C ha^−1^ and linearly increased with latitude. As a fast-growing plant, Moso bamboo could be harvested amounts of 6.0 Mg C ha^−1^ to 7.6 Mg C ha^−1^ annually, which indicates a high potential of this species for carbon sequestration. Parameters obtained in this study can be used to accurately estimate the carbon stock of Moso bamboo forest to establish models of the global carbon balance.

## Introduction

Bamboo (*Bambuseae*), an important forest type worldwide, is mostly distributed in the tropical and subtropical regions of Asia. In China, the bamboo area is approximately 6.01 × 10^6^ ha and accounts for approximately 3% of the total forest area [1]. Bamboo provides wood and food for human consumption and presents economic and ecological benefits [2, 3]. The growth patterns of bamboos differ from timber, and their unique characteristics include fast growth, high production, and rapid maturation from shoots to culms [2, 4]. About 300 species of bamboo from 44 genera are available in China, and *Phyllostachys pubescens* (Moso bamboo) forest occurs extensively (4.20 × 10^6^ ha) and dominates bamboo forests (70% of total bamboo cover) [1]. Moso bamboo is a large bamboo species and harvested for both poles and edible shoots throughout South East Asia. Bamboo culms are sprouted by horizontal rhizome systems and present a remarkable productivity [2, 4, 5]. Both rhizomes and culms are produced underground every two years asynchronously. The age of mature culm is 10 years and it is harvested usually at 6–8 years. Thus, bamboo forests are unevenly aged stands, where differently aged culms are distributed within a stand [2, 6].

Terrestrial forest forests play an important role in the global carbon cycle and present a potential in mitigating the warming effect through carbon sequestration [7, 8]. Many studies have focused on the contributions of large woody plants to carbon stock, but very few studies have focused on bamboo plants [9–14]. Fast-growing Moso bamboo has a high production and potential in carbon sequestration. Estimation of Moso bamboo carbon stock considerably varies [15–19], which could be attributed to exclusion of several influencing factors because of limited available data. Moso bamboo is distributed extensively in China, and its natural habitat region extends approximately between 23°30′ N to 32°20′ N and 104°30′ E to 122° E [4]. However, no data are available regarding variations in the carbon stock of Moso bamboo with latitude gradient, which is an important parameter in global carbon sequestration evaluation of terrestrial forests. Therefore, the objectives of this study were: (1) to estimate change trends in the biomass and carbon stock of Moso bamboo in various sites in relation to latitude, (2) to determine the annual yield and potential of Moso bamboo in carbon fixation, and (3) to establish valuable parameters for extensive carbon sequestration estimation models.

## Materials and methods

### Sampling site description

**Fig 1:**
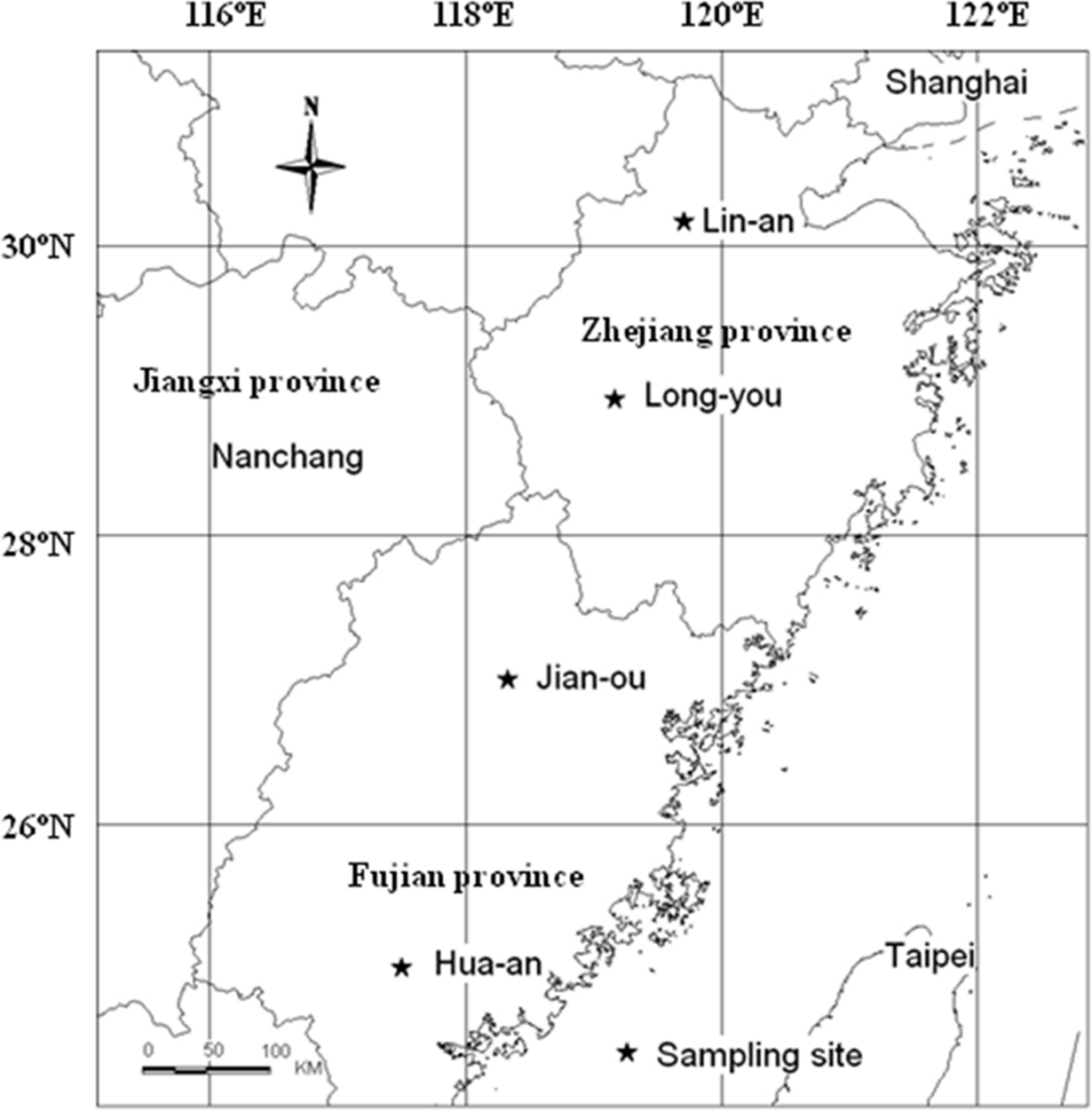
Location of the study sites.

In China, Moso bamboo is mostly distributed in the provinces of Zhejiang, Fujian, Jiangxi, and Hunan. The central forest area is situated between 25° N to 30° N and 110° E to 120° E [4]. We selected four typical and representative counties from north to south within the central area (Fig 1). These counties included Lin-an, Long-you, Jian-ou, and Hua-an. The areas of Moso bamboo in these four counties are 4.2×10^4^, 1.6×10^4^, 8.0×10^4^ and 1.2×10^4^ ha in 2013, respectively. There was no specific permissions were required for these locations. All sites in this study are common for bamboo shoot and culm production. The field studies did not involve endangered or protected species. In order to minimize the artificial factors, the selected bamboo plots were managed as the same mode, i.e. which were harvested for culm and shoot without fertilizer and pesticide application. According to the survey, the basic related characteristics of these sites are listed in Table 1. The difference of latitude from north to south is 5.16°, with a distance of 560 km. The elevations of sampling site range from 165 m to 278 m above sea level. The mean temperature of these sites increases from north to south with increasing latitude, and the annual mean rainfall in all sites is around 1600 mm. All selected sites belong to a typical monsoon region. Zhejiang and Fujian provinces are located in the red soil region in China, in which red soil is extensively present. The soil type of the selected bamboo sites is red-yellowish forest soil.

**Table 1:** Basic information of the sampling sites.

Sampling site	Longitude (°)	Latitude (°)	Elevation (m)	Temperature (°C)	Rainfall (mm)
**Lin-an**	119.71	30.18	165–220	16.4	1628
**Long-you**	119.16	28.94	248–265	17.1	1602
**Jian-ou**	118.33	27.01	250–278	19.3	1670
**Hua-an**	117.49	25.02	204–268	21.0	1618

### Sampling method

Bamboo survey was conducted in September 2013 in Lin-an, Long-you, Jian-ou, and Hua-an counties. In each county, three pure Moso bamboo forests with a similar elevation were selected. In each site, three bamboo plots were chosen and assigned as 10 m × 10 m that is the minimum area of the national survey requirement. In total, 36 plots were involved in this survey. We recorded the age and the diameter at breast height (DBH) of each bamboo culm in each plot in situ. Bamboo age was identified as described by [4]. In brief, (1) for bamboos of 1–2 years, culm color is dark green, the eyelash on culm sheath cycle is brown, and the powder under culm sheath cycle is white with sheaths in the culm base. (2) For bamboos of 3–4 years, culm color is green, the eyelash on culm sheath cycle is sparse, and the powder under culm sheath cycle is grayish. (3) For bamboos of 5–6 years, culm color is yellowish green and the powder under culm sheath cycle is grayish black with a wax layer on the culm. (4) For bamboos of 7–8 years, culm color is greenish yellow with a wax layer on the culm. (5) For bamboos of 9–10 years, culm color is bronze with a wax layer that begins to fall. In order to verify the bamboo biomass estimation equation, 9 single bamboos in each site were randomly collected from root to leaf. The bamboo plant was divided into root, rhizome, stump, culm and leaf/stick. Each part of every bamboo plant was weighted and sampled in situ and taken back to lab for water and C analysis.

Given that bamboo is a shallow-rooted plant, with its roots generally concentrated in the upper 40 cm of soil [17], we collected soil samples up to 60 cm depth. The soil sample layers were divided into four: 0–10, 10–20, 20–40, and 40–60 cm. Soil bulk density was measured simultaneously through soil core–ring method in each layer. Litter above the ground was also collected in three 1 m × 1 m quadrats per plot.

### Sample analysis

Organic carbon in bamboos and litters was determined with Elemental Analyzer (Vario-MAX, Germany). Soil organic matter was measured through K_2_CrO_4_ oxidation method [20]. Soil bulk density was calculated based on mass weight [21]. Dry biomass of bamboos was obtained after deduction of water content.

### Computation and statistics

A single Moso bamboo biomass is cumulated from various bamboo sections and the total bamboo biomass in each plot can be obtained by the sum of all bamboos. Considering the limited number of measured bamboos (total 36 culms), we adopted an empirical equation to estimate bamboo biomass in each plot.

$$M=747.787D^{2.771}{\left[0.148A/\left(0.074728+A\right)\right]}^{5.555}+3.772$$ (1)

where *M* is the biomass of single bamboo plant, kg; *D* is the DBH of bamboo, cm; and *A* is the “Du” of bamboo related to bamboo age [15]. In China, the age of Moso bamboo was recorded in “Du”, showing the growth habit in “on” and “off year” bamboo stands. In particular, 1 “Du” corresponds to 1–2 years of age, whereas 2, 3, and 4 “Du” are 3–4, 5–6, and 7–8 years old, respectively [4]. Parameters of the Eq 1 were verified by the 36 measured bamboos. Results showed that the calculated and proposed parameters had no significant difference, thereby suggesting that the Eq 1 was suitable in this study. Accordingly, C stock of bamboo biomass in each plot was cumulated as Mg C ha^−1^ with each bamboo biomass and C concentration.

Soil organic carbon (SOC) stock was estimated based on the area of bamboo stands and SOC stocks per hectare:

$$SOC=0.58\cdot \sum SD_{i}SOM_{i}D_{i}\times 10^{2},$$ (2)

where 0.58 is the coefficient transformed from soil organic matter to *SOC*; *SD*_*i*_ represents soil bulk density in each layer; *SOM*_*i*_ is the content of soil organic matter in the layer; and *D*_*i*_ is the thickness of layer depths, i.e., 10, 10, 20, and 20 cm. Carbon stock in bamboo stands was cumulated with biomass carbon, soil carbon, and litter carbon.

The bamboo DBH distribution pattern was described using Weibull model. The model equation is: $f(x)=\frac{c}{b}{\left(\frac{x-a}{b}\right)}^{c-1}\times e^{-{\left(\frac{x-a}{b}\right)}^{c}},x>a$, where a, b, c are parameters of the survival function. The simulation of bamboo DBH with Weibull distribution and normal distribution was carried out using Origin software (Origin 8.6).

The annual carbon sequestration rate of Moso bamboo was calculated as the part of biomass removed annually. According to the field survey, the distribution ratio of Moso bamboo age was 1:1:1:1 on the basis of “Du”, indicating that 1/4 of the bamboo forest was harvested every 2 years. Therefore, the annual carbon sequestration rate was equal to 1/8 of the stand biomass.

The data obtained in the survey was analyzed by SPSS software (SPSS 20.0). The difference of data in various groups of Moso bamboo was tested by LSD method with a level at 0.05 (marked as letters).

## Results

### Bamboo biomass varied with latitude

**Fig 2:**
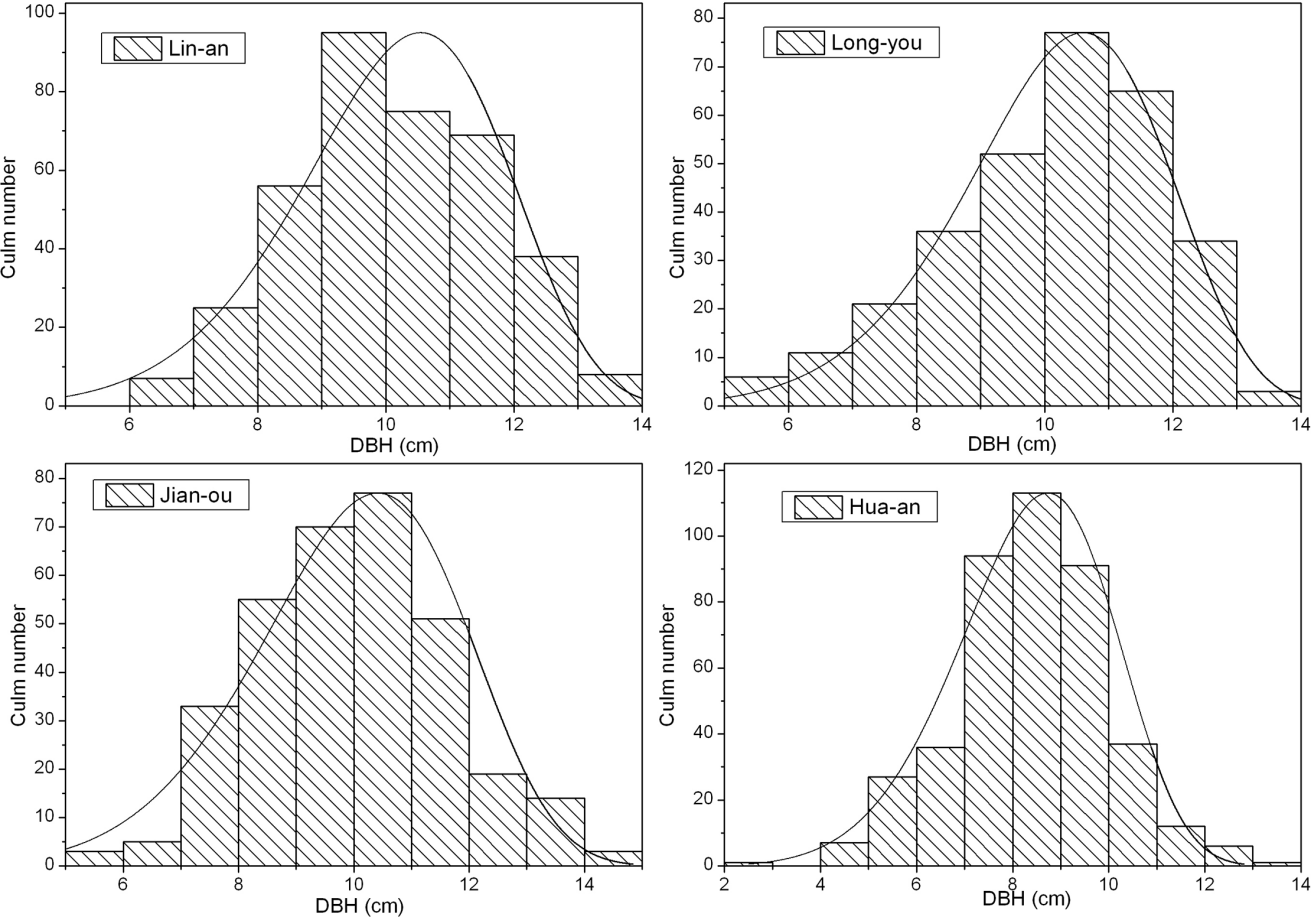
Diameter at breast height (DBH) distribution of Moso bamboo in various sites.

As shown in Table 2, the average DBH of Moso bamboo stands ranged from 8.37 cm to 10.12 cm and decreased with decreasing latitude. The DBH distribution pattern in various sites in the probability function is shown in Fig 2. Table 2 showed the parameters of DBH distribution in Moso bamboo stands, which were fitted with normal and Weibull distribution models. The goodness-of-fit test results (*R* value) showed that the DBH of Moso bamboo in these sites could be fitted well with Weibull rather than with normal distribution model; hence, the Weibull distribution model has a potential in predicting Moso bamboo biomass and carbon stock.

**Table 2:** Characteristics of DBH distribution in Moso bamboo stands obtained with normal and Weibull distribution models (cm).

Site	DBH	Normal			Weibull		
	Average	Mean	SD	R	a	b	R
**Lin-an**	10.10	10.10	1.55	0.895	10.77	7.13	0.912
**Long-you**	10.12	10.12	1.69	0.867	10.81	7.32	0.935
**Jian-ou**	9.99	9.99	1.68	0.872	10.70	6.36	0.954
**Hua-an**	8.37	8.37	1.57	0.893	9.01	5.90	0.923

According to the field plot survey, the bamboo culm densities were 3400 ± 510, 3378 ± 353, 3667 ± 628, and 4722 ± 1065 culm ha^−1^ in Lin-an, Long-you, Jian-ou, and Hua-an site, respectively. Bamboo culm densities did not significantly differ among these sites but showed an increasing trend with decreasing latitude, that is, the culm density increased from north to south. This trend was consistent with that of bamboo DBH, in which a stand containing several culms showed a low average DBH. Due to the similar condition, temperature could be responsible for such a trend of DBH.

As revealed in Table 3, the bamboo biomass mainly occurred in culm that ranged from 45.15 to 58.26% of the total. It was the lowest in the litter on ground from 1.85 to 3.26%. The biomass carbon distribution pattern was not significantly different among various sites. The biomass carbon percentage of various sections showed an order as: culm > root > leaf/stick > stump > rhizome > litter. When the bamboo harvested, the residues in soil included root, rhizome, stump and litter that could be 35.1%, suggesting a high soil C sequestration potential of bamboos.

**Table 3:** Biomass carbon distribution in various sections of single bamboo.

Site	Leaf/stick (%)	Culm (%)	Root (%)	Rhizome (%)	Stump (%)	Litter (%)
**Lin-an**	11.56 b	45.15 a	13.73 a	8.18 a	18.11 a	2.97 a
**Long-you**	16.96 ab	48.72 a	11.23 a	7.73 a	13.47 a	1.85 a
**Jian-ou**	13.84 b	58.26 ab	9.28 a	5.15 a	11.44 a	2.08 a
**Hua-an**	14.40 b	50.44 a	14.82 ab	7.30 a	9.83 a	3.26 a
**Mean**	14.19	50.64	12.27	7.09	13.21	2.54

Bamboo biomass carbon was calculated with Eq 1 by using the results of DBH and age of bamboos. As listed in Table 4, the bamboo biomass was 60.58 ± 15.6, 49.07 ± 8.29, 52.67 ± 6.73, and 48.31 ± 12.4 Mg C ha^−1^ in Lin-an, Long-you, Jian-ou and Hua-an, respectively. The bamboo biomass decreased from north to south, but the difference level at 5% was not significant.

**Table 4:** Moso bamboo biomass in various sites (Mg C ha^−1^).

Site	Plot1	Plot2	Plot3	Plot4	Plot5	Plot6	Plot7	Plot8	Plot9	Average
**Lin-an**	52.60	49.44	45.55	35.72	72.14	67.27	66.77	85.30	70.45	60.58 ± 15.60 a
**Long-you**	57.48	56.81	50.44	44.34	62.83	41.25	40.88	40.53	47.11	49.07 ± 8.29 b
**Jian-ou**	51.79	47.63	42.62	55.86	49.73	50.27	50.49	63.45	62.16	52.67 ± 6.73 ab
**Hua-an**	48.28	41.51	44.92	59.91	28.19	45.44	47.06	73.16	46.30	48.31 ± 12.40 b

### Soil carbon stock of Moso bamboo forests

**Fig 3:**
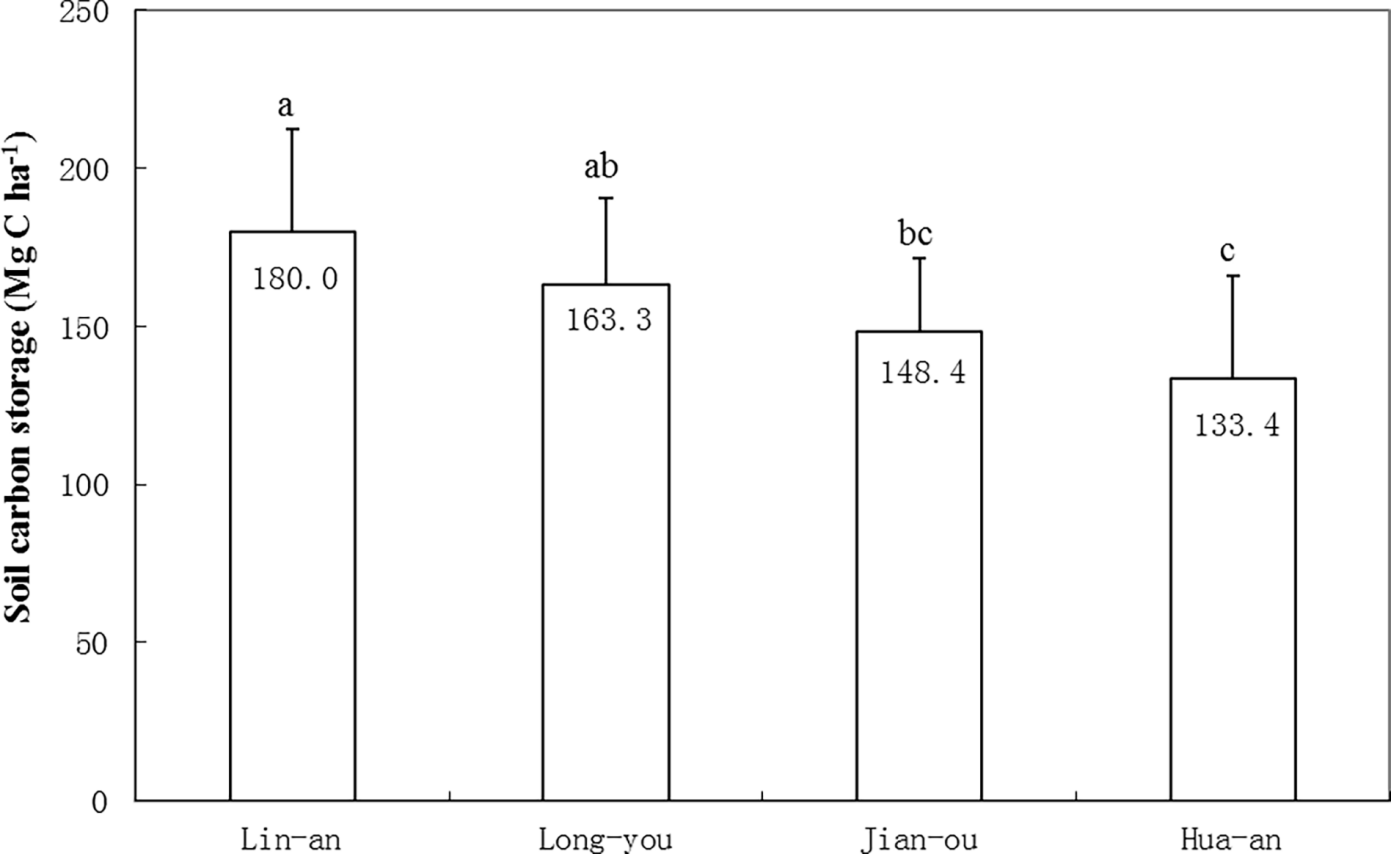
Carbon stock in Moso bamboo forest in a latitude sequence.

The soil carbon stock of Moso bamboo forest was calculated from 0 to 60 cm with soil bulk density and SOC content. The results showed that soil bulk density increased with increasing soil depth, but SOC content presented an opposite trend. Soil carbon stock was the highest in the layer of 20–40 cm. The total soil carbon stock in 0–60 cm layers was 119.5 ± 16.7, 114.7 ± 18.9, 98.2 ± 16.2, and 87.83 ± 20.1 Mg C ha^−1^ in the four sites from north to south (Table 5). The decreasing trend of soil carbon stock from north to south in Moso bamboo forest was obvious, and the difference was significant at 5% level. Based on the results of bamboo biomass and soil carbon stocks, total carbon stock in Moso bamboo forests was obtained as 180.01 Mg C ha^−1^ in Lin-an and 133.41 Mg C ha^−1^ in Hua-an (Fig 3). The decrease rate in carbon stock was significantly linear (*R*^2^ = 0.983) from north to south by a step of 7.77 Mg C ha^−1^ per 100 km. Moreover, the percentage of bamboo biomass carbon in the total carbon stock ranged from 30% to 36%. The biomass percentage increased from north to south, thereby suggesting a relatively higher biomass production in low latitudes than soil carbon sequestration.

**Table 5:** Soil bulk density and organic matter content in layers of Moso bamboo soil (*n* = 9).

	Soil layer (cm)	Site			
		Lin-an	Long-you	Jian-ou	Hua-an
**Bulk density (g cm**^**−3**^**)**	0–10	1.122 ± 0.205	1.070 ± 0.065	0.980 ± 0.199	0.936 ± 0.155
	10–20	1.128 ± 0.231	1.092 ± 0.128	1.014 ± 0.188	1.039 ± 0.153
	20–40	1.183 ± 0.251	1.169 ± 0.074	1.058 ± 0.124	1.182 ± 0.216
	40–60	1.201 ± 0.212	1.146 ± 0.102	1.149 ± 0.118	1.283 ± 0.128
**Soil organic matter (g kg**^**−1**^**)**	0–10	44.13 ± 25.60	41.91 ± 7.79	41.14 ± 12.53	40.07 ± 12.2
	10–20	34.18 ± 19.83	33.57 ± 8.22	32.41 ± 9.77	29.34 ± 6.90
	20–40	28.42 ± 16.49	27.34 ± 7.25	26.69 ± 9.38	20.69 ± 9.32
	40–60	23.92 ± 10.21	22.87 ± 6.02	17.23 ± 4.99	13.46 ± 6.55
**Soil carbon stock (Mg C ha**^**−1**^**)**	0–10	26.87 ± 12.8	26.02 ± 4.72	23.39 ± 3.83	21.75 ± 5.00
	10–20	21.54 ± 4.84	21.26 ± 3.83	19.05 ± 4.28	17.68 ± 4.49
	20–40	37.77 ± 12.80	37.09 ± 9.26	32.75 ± 11.10	28.36 ± 9.62
	40–60	33.33 ± 6.85	30.40 ± 8.41	22.96 ± 6.00	20.04 ± 8.86
**Total**	0–60	119.5 ± 16.7 a	114.7 ± 18.9 b	98.2 ± 16.2 c	87.83 ± 20.1 d

## Discussion

The diameter at breast height (DBH) is one of the most important parameters for underground or aboveground biomass estimation [22, 23]. Similarly, DBH was also used in the estimation of mankino bamboo biomass [24] and Moso bamboo [25]. In this study, the DBH distribution pattern of Moso bamboo was fitted well by Weibull models, suggesting that DBH parameter obtained in this study would be useful for other models.

The recorded density of Moso bamboo culm ranged from 1350 culm ha^−1^ to 4545 culm ha^−1^ [15], and the present results, which ranged from 3378 culm ha^−1^ to 4722 culm ha^−1^, mostly fell into the scale. The bamboo culm density in our investigation was considerably higher because bamboo farmers enforced bamboo management and removed bamboo culms not in complete maturation, thereby making the percentage of low age increased in the stand. In this study, there was no 8-year-old bamboo observed and, in particular, the bamboo age was even less than 6 years old in Hua-an site. Comparatively, the mature bamboo age is usually older than 10 years in natural bamboo stands [4]. The bamboo culm removal in younger age might be responsible for a high culm density with a small DBH.

The biomass of Moso bamboo ranged from 23.7 t d. m. ha^−1^ to 572.3 t d. m. ha^−1^, which is equal to 11.8 Mg C ha^−1^ to 286.2 Mg C ha^−1^ as previously reported [15, 19]. We speculated that some reports overestimated Moso bamboo biomass. Yen et al. [14] estimated that the annual carbon sequestration rate in Moso bamboo was 8.13 Mg C ha^−1^ yr^−1^. Similarly, the rate of aboveground C sequestration was estimated to be 18.93–23.55 Mg C ha^−1^ yr^−1^ with a mean of 21.36 Mg C ha^−1^ yr^−1^ in northeast India [26]. As evaluated by Keith et al. [8], the default total biomass was 132 Mg C ha^−1^ to 171 Mg C ha^−1^ in subtropical forests. Obviously, the biomass of Moso bamboo stands was relatively lower than the data available. However, Moso bamboo is a fast-growing plant with a short term of harvest within 5 to 10 years. According to the present investigation, the distribution ratio of Moso bamboo age was 1:1:1:1 on the basis of “Du.” This finding means that 1/4 of the bamboo forest was harvested every 2 years. Thus, the harvested bamboo biomass ranged from 6.0 Mg C ha^−1^ to 7.6 Mg C ha^−1^ annually. This carbon fixation rate was higher by 2.52 times than that of a fast-growing *Cunninghamia lanceolata* [27] and higher by 3.73 times than that of a *Pinus taeda* plantation [28]. This finding suggested that Moso bamboo has a high potential in carbon fixation and it could be a good candidate species for carbon stock in this region.

Soil organic carbon (SOC) is the most important carbon pool on the global scale [29, 30]. The estimated global SOC pool is 1550 Pg C, which is twice higher than that in the atmosphere (770 Pg C) and 2.5 times higher than that in the biotic pool (610 Pg C) [31]. Accordingly, any change in the size and turnover of SOC pools may potentially alter the atmospheric CO_2_ concentration and the global climate [32, 33]. The pathway to increase SOC is critical to mitigate global warming effect. In this study, soil carbon stock within 0–60 cm layer of Moso bamboo stands ranged from 87.83 Mg C ha^−1^ to 119.5 Mg C ha^−1^ (Fig 3), with an average of 103.6 Mg C ha^−1^, which is significantly higher than that in paddy (69.24 Mg C ha^−1^) and upland (49.91 Mg C ha^−1^) soils in China [34]; this average amount is also even higher than the average forest stock (97.8 Mg C ha^−1^). As shown in Table 3, a high ratio of belowground of bamboos could be one important factor resulting in a high C sequestration in soil. Therefore, Moso bamboo forest may play an important role in effective CO_2_ sequestration.

**Fig 4:**
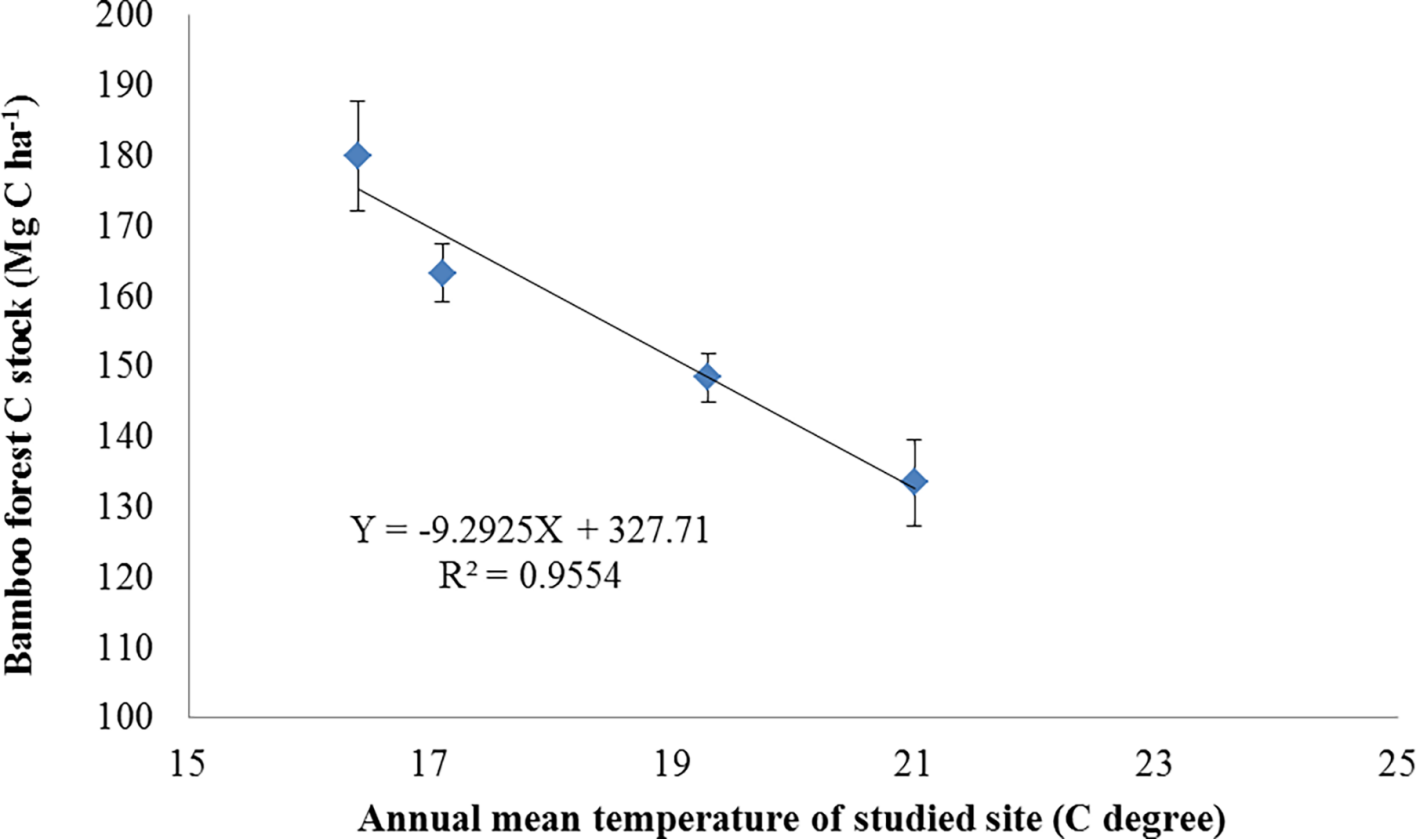
Relationship between Moso bamboo forest C stock and annual mean temperature.

Latitudinal zonality is usually consistent with climate and vegetation zonality. In this study, latitude determines the local temperature that increases from north to south (Table 1). Temperature influences plant photosynthesis and respiration, which significantly affect the carbon balance in terrestrial ecosystems. Many studies showed that soil organic matter decreases with temperature [35–39]. In this study, we selected bamboo sites with a similar elevation and annual rainfall, considering that temperature is mainly responsible for the difference of carbon stocks in Moso bamboo forests. The regression analysis results showed that carbon stock in Moso bamboo forests was linear and negatively correlated with the local mean temperature (*R*^2^ = 0.955, Fig 4). However, the relationship between bamboo biomass carbon and temperature was not significant, thereby suggesting that manual harvest practices exerted an important influence on bamboo stands. In particular, a different management practice can result in various bamboo age distributions. Moreover, the decrease trend of soil C stock with latitude in Moso bamboo forest was confined within the region with similar altitude and precipitation. The parameter of bamboo forest C change with latitude was only suitable for this region when used in a model, but it could be used as a reference to other regions.

An accurate or realistic estimation of carbon stock is important for a comprehensive evaluation of Moso bamboo in carbon sequestration and ecological function. Our results provided a useful data set for carbon stock estimation of Moso bamboo forests in a large scale and a suitable estimation model.

## Conclusions

A field survey with 36 plots of Moso bamboo forests along a latitude gradient was carried out. Results showed that DBH of Moso bamboo culms increased from 8.37 cm to 10.12 cm, whereas the bamboo culm density decreased from 4722 culm ha^−1^ to 3400 culm ha^−1^ with increasing latitude. The bamboo biomass decreased from 60.58 Mg C ha^−1^ to 48.31 Mg C ha^−1^ from north to south. The total carbon stock of Moso bamboo forests, which comprises soil and biomass carbon, ranged from 87.83 Mg C ha^−1^ to 119.5 Mg C ha^−1^ and linearly increased with latitude. The carbon sequestration rate of Moso bamboo ranged from 6.0 Mg C ha^−1^ to 7.6 Mg C ha^−1^ annually, suggesting it is a candidate species for carbon fixation in the study region. Parameters obtained in this study can be used to accurately estimate the carbon stock of Moso bamboo to establish models of the global carbon balance.
